# Allosteric modulation of GABA_A_ receptors by extracellular ATP

**DOI:** 10.1186/1756-6606-7-6

**Published:** 2014-01-24

**Authors:** Jun Liu, Yu Tian Wang

**Affiliations:** 1 Brain Research Centre and Department of Medicine, Vancouver Coastal Health Research Institute, University of British Columbia, Vancouver, BC V6T 2B5, Canada; 2 Translational Medicine Research Center, China Medical University Hospital, Graduate Institute of Immunology, China Medical University, Taichung 40447, Taiwan; 3 Current address: Medical Research Center, Qianfoshan Hospital, Shandong University, Jinan, Shandong 250014, China

**Keywords:** GABA_A_ receptor, ATP, Allosteric potentiation, Neuronal cultures

## Abstract

**Background:**

The γ-aminobutyric acid type A receptor (GABA_A_R) is the primary receptor mediating fast synaptic inhibition in the brain and plays a critical role in modulation of neuronal excitability and neural networks. Previous studies have demonstrated that ATP and its nucleotide analogs may regulate the function of GABA_A_Rs via Ca^2+^-dependent intracellular mechanisms, which require activation of purinergic 2 (P2) receptors or cross-talk between two receptors.

**Results:**

Here, we report a potentiation of GABA_A_Rs by extracellular ATP via a previously un-recognized allosteric mechanism. Using cultured hippocampal neurons as well as HEK293 cells transiently expressing GABA_A_Rs, we demonstrate that extracellular ATP potentiates GABA_A_R mediated currents in a dose-dependent manner with an EC50 of 2.1 ± 0.2 mM. The potentiation was mediated by a postsynaptic mechanism that was not dependent on activation of either ecto-protein kinase or P2 receptors. Single channel recordings from cell-free excised membrane patches under outside-out mode or isolated membrane patches under cell-attached mode suggest that the ATP modulation of GABA currents is achieved through a direct action of ATP on the channels themselves and manifested by increasing the single channel open probability without alteration of its conductance. Moreover, this ATP potentiation of GABA_A_R could be reconstituted in HEK293 cells that transiently expressed recombinant rat GABA_A_Rs.

**Conclusions:**

Our data strongly suggest that extracellular ATP allosterically potentiates GABA_A_R-gated chloride channels. This novel mode of ATP-mediated modulation of GABA_A_Rs may play an important role in regulating neuronal excitability and thereby in fine-tuning the excitation-inhibition balance under conditions where a high level of extracellular ATP is ensured.

## Background

The γ-aminobutyric acid type A receptor (GABA_A_R) is a ligand-gated chloride ion channel, activation of which results in membrane hyperpolarization and hence inhibition of the neuronal excitability in the adult mammalian brain. Dysfunction of GABA_A_Rs is associated with the pathogenesis of a number of neurological diseases and neuropsychiatric disorders such as epilepsy, Alzheimer disease, and anxiety [1-6]. GABA_A_Rs are also targets of many clinically-relevant drugs including benzodiazepine, barbiturates and general anesthetics [7]. Moreover, endogenously produced substances such as neurosteroids [8] and zinc [9,10] modulate GABA_A_Rs via direct interaction with the putative binding sites on the receptor subunit. Therefore, allosteric modulation is an important mode in regulating GABA_A_R functions and hence maintaining homeostasis for neuronal excitability.

In the CNS, adenosine 5′-triphosphate (ATP) not only acts as a major intracellular energy source and phosphate donor, but also functions extracellularly as a neurotransmitter via activation of purinergic 2 (P2) receptors. Previous studies demonstrate that extracellular ATP can modulate GABA_A_R function by activation of P2 receptors [11-13]. In addition, a physical cross-talk between GABA_A_Rs and P2 receptors which influencess inhibition of GABA_A_R-mediated currents has also recently been reported [14-16]. Ortinau et al. (2003) reported that extracellular ATP inhibits the function of N-methyl-D-aspartate (NMDA) glutamate receptors by directly binding to the receptor, suggesting that extracellular ATP may function as an allosteric modulator for neurotransmitter receptors [17].

High levels of ATP also exist in the extracellular compartment under both normal physiological conditions (i.e. as result of synaptic release) [18-20], and pathological conditions such as traumatic and ischemic brain insults [21-24]. In addition, previous studies suggest that ATP and GABA are released at GABAergic synapses [18,19,25]. Such a co-release suggests that, under certain conditions, ATP could act as an allosteric modulator for postsynaptic GABA_A_Rs. In the current study, we set out to investigate this hypothesis by using both cultured hippocampal neurons and HEK293 cells transiently expressing functional recombinant GABA_A_Rs. We found that both ATP and ADP can potentiate GABA_A_R-mediated currents. Moreover, this potentiation effect appears to be mediated by a direct binding of these nucleotides at a putative nucleotide-binding site on the GABA_A_R.

## Results

### Extracellular ATP potentiates GABA_A_R-mediated currents

**Figure 1:**
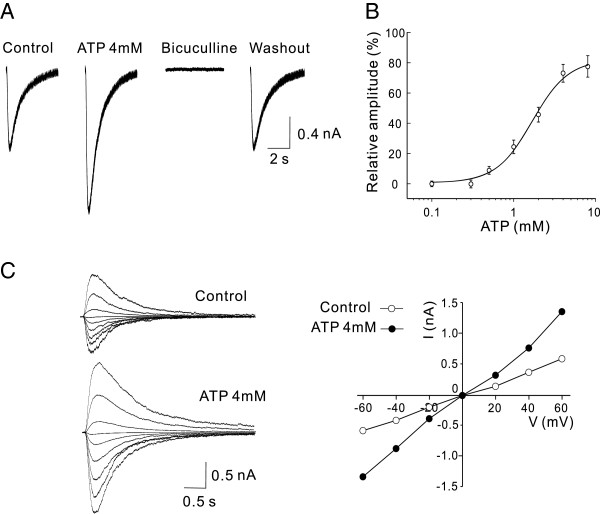
**Extracellular ATP dose-dependently potentiates GABA**_**A**_**R-mediated currents in neurons.** Whole-cell patch-clamp recording were made from rat cultured hippocampal neurons. GABA_A_R-mediated currents were evoked by a pulse pressure ejection of GABA (100 μM) from the tip of a pipette positioned close to the neurons under recording. **A**, Bath application of ATP (4 mM) reversibly potentiates GABA currents (ATP; 171.9 ± 13.8% of the Control; P < 0.01; n = 7) and the potentiated currents remained sensitive to bicuculline (10 μM) blockade (ATP + bicuculline). Representative current traces were taken 5 min before and after drug applications or washout as indicated. **B**, The dose-response curve showing the dose-dependent potentiation of GABA currents by various concentrations of ATP (n = 8). Each data point represents the mean ± SEM of GABA currents (normalized to Control) at the indicated ATP concentrations. The solid line is the best fit of the data to the Hill equation, which yields a mean EC_50_ of 2.1 ± 0.3 mM and H of 1.66. **C**, ATP increases the amplitude of GABA currents without altering their reversal potential. *Left:* Superimposed individual traces of currents evoked by GABA (10 μM) in the absence (Control) and presence of ATP (4 mM) at different holding potentials from -60 to +60 mV with a step of 20 mV. *Right*: current-voltage (I-V) relationships constructed from the data shown in the *Left*.

Under whole-cell voltage-clamp recordings of hippocampal neurons at a holding membrane potential of -60 mV, repetitive short pulses of pressure ejections of GABA (100 μM) to the neuron evoked robust inward currents, and these currents were mediated through GABA_A_R-gated Cl^-^ channels as they were completely blocked by bath application of the GABA_A_R antagonist bicuculline (10 μM; Figure 1A). Bath application of ATP at various concentrations produced a dose-dependent potentiation of the GABA-evoked currents with an EC50 of 2.1 ± 0.2 mM (n = 8; Figure 1A and B). At a concentration of 4 mM, ATP increased the GABA-evoked currents to 171.9 ± 13.8% of the control (n = 7, P < 0.01). The potentiation of ATP on GABA currents was reversible upon washout and the potentiated currents remained sensitive to bicuculliine blockade (Figure 1A). Analysis of the current-voltage (I - V) relationship in the absence and presence of ATP within the range of holding potentials from -60 to +60 mV showed that ATP significantly potentiated the peak amplitude of GABA currents without altering the reversal potential (Figure 1C). Thus, extracellular ATP at mM ranges can significantly potentiate the function of GABA_A_Rs.

### Extracellular ATP potentiates currents mediated by both synaptic and extrasynaptic GABA_A_Rs

**Figure 2:**
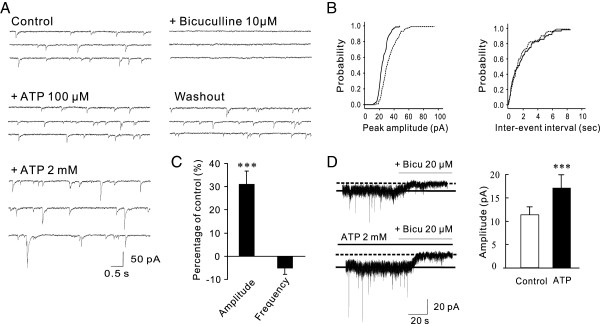
**Extracellular ATP potentiates function of both synaptic and extrasynaptic GABA**_**A**_**Rs in cultured hippocampal neurons. A-C**, ATP increases the amplitude (but not the frequency) of mIPSCs mediated by synaptic GABA_A_Rs. Pharmacologically isolated GABA_A_R-mediated mIPSCs were recorded under whole-cell voltage clamp mode at a holding potential of -60 mV. **A**, Representative continuous recording traces taken 5 min before and after drug applications or washout showing dose-dependent effects of ATP on mIPSCs. ATP at a concentration of 2 mM, but not 100 μM, potentiated mIPSCs. The potentiated mIPSCs were fully blocked by additional application of bicuculline (10 μM) and returned to the control level after ATP washout. **B**, Cumulative amplitude and frequency distribution histograms of mIPSCs from the same cell shown in **A** before (black lines) and after (dotted lines) ATP application illustrating a specific increase in the mIPSC amplitude without alteration of its frequency. **C**, Histogram summarizing averaged changes in the amplitude and frequency of mIPSCs before (Control) and after application of ATP (2 mM) from 7 individual neurons. All values were presented as percentage of the controls. **D**, Extracellular ATP increases the tonic GABA current. Tonic GABA currents were revealed by blocking native GABA_A_Rs with addition of bicuculline (20 μM) in the absence (Control) and presence (ATP 2 mM) of ATP in the extracellular recording solution. *Left*, Representative tonic GABA current traces in the absence and presence of ATP taken from the same neuron 5 min before and after bath application of ATP (2 mM). Bar graphs on the *Right* summarizing data obtained from 6 individual neurons. ** P < 0.01.

Currents evoked by exogenously applied GABA could be mediated by synaptic and/or extrasynaptic GABA_A_Rs. To examine if ATP can modulate GABA_A_Rs localized at the synapse, we examined effects of extracellularly applied ATP on whole-cell recordings of miniature postsynaptic inhibitory currents (mIPSCs) mediated by synaptic GABA_A_Rs. mIPSCs were recorded at a holding membrane potential of -60 mV after blocking ionotropic glutamate receptors with CNQX (20 μM) and AP-5 (50 μM), glycine receptor with strychnine (1 μM) and sodium channels with TTX (1 μM). Suramin (100 μM) and BBG (1 μM) were also added to the extracellular solution to block P2 receptors. Similar to the observation with evoked GABA currents above, extracellular application of ATP at a concentration of 2 mM (but not 100 μM) significantly potentiated mIPSCs (Figure 2A and B). These increased mIPSCs were abolished by addition of bicuculline (10 μM), indicating that mIPSCs following ATP remained entirely gated through GABA_A_Rs (Figure 2A). The ATP enhancement of mIPSCs was reversible, as it recovered to control levels after ATP and bicuculline wash out (Figure 2A). Consistent with an effect on the postsynaptic GABA_A_R, but not on presynaptic GABA release, ATP potentiation was manifested as a specific increase in the amplitude (132 ± 8.7% of the control; p < 0.01; n = 7; Figure 2E), but not the frequency (96.4 ± 3.3% of the control; p > 0.05; Figure 2E), of mIPSCs. These results indicate that extracellular ATP potentiates function of synaptic GABA_A_Rs, thereby increasing the synaptic (phasic) currents mediated by these receptors.

Next, to determine if ATP has a modulatory effect on extrasynaptic GABA_A_Rs, thereby affecting tonic GABA currents, we examined tonic GABA currents revealed by the addition of bicuculline (20 μM) in the presence or absence of ATP (2 mM ATP). As shown in Figure 2D, bath application of bicuculline (20 μM) produced an outward shift of the baseline current trace, indicating that these tonic currents are gated largely through extrasynaptically localized GABA_A_Rs activated by ambient GABA under the recording conditions [26]. Following addition of ATP (2 mM), bicuculline produced a significantly larger outward shift of the holding current (Figure 2D; 17.2 ± 2.7 pA in the presence of ATP vs 11.4 ± 1.8 pA in the absence of ATP; P < 0.01; n =6). Thus, extracellular ATP appears capable of modulating both synaptic and extrasynaptic GABA_A_Rs, thereby potentiating both phasic and tonic GABA currents.

Extracellular ATP modulates GABA_A_R function via mechanism of independent of ecto-protein kinases or activation of P2 receptors.

**Figure 3:**
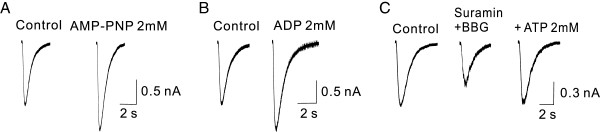
**Potentiation of GABA**_**A**_**R-mediated currents by ATP and analogs does not require activation of ecto-protein kinase or P2 receptors in cultured hippocampal neurons.** Representative individual whole-cell GABA current traces were taken 5 min before (Control) and after drug applications (100 μM, pulse-pressure ejection). **A** and **B**, AMP-PNP (2 mM; **A**) or ADP (2 mM; **B**), two ATP analogues that cannot function as phosphate donor for ecto-protein kinase mediated phosphorylation, remain capable of potentiating GABA currents, increasing the peak currents by 134.2 ± 11.4% (**A**; P < 0.05; n = 5) and 140 ± 14.7 (**B**; P < 0.05; n = 7), respectively. **C**, Non selective P2 receptor antagonist suramin (100 μM) and selective P2X_7_ receptor antagonist BBG (1 μM) suppressed basal GABA currents on their own, but failed to prevent ATP from potentiating the currents; ATP increased the peak currents by 138.5 ± 16.5% (n = 5; P < 0.05) in the presence of both antagonists.

Intracellular ATP is usually maintained at a few millimolar range (2-5 mM) at which it serves as a phosphate donor to support regulation of GABA_A_R by protein phosphorylation [27-29]. As extracellular (ecto-) protein kinases have been previously demonstrated to phosphorylate neuronal membrane proteins such as P2X3 receptors [30], we first examined the possibility that the ATP potentiation of GABA_A_R function was a result of ecto-protein kinase mediated phosphorylation by using AMP-PNP, a non-hydrolyzable analog of ATP that cannot support the protein phosphorylation process. As illustrated in Figure 3A, AMP-PNP (2 mM) in the bath mimicked ATP, increasing the amplitude of GABA currents (Figure 3A; 134.2 ± 11.4%; P < 0.05; n = 5). Similarly, we found that ADP (2 mM) was also able to significantly potentiate GABA currents (Figure 3A; 140 ± 14.7; P < 0.05; n = 7). As both AMP-PNP and ADP cannot function as a phosphate donor during protein phosphorylation process, these results suggest that extracellular protein phosphorylation is not likely involved in the ATP potentiation of GABA_A_R-mediated currents.

As a neurotransmitter, at low concentrations (<100 μM) ATP can activate a number of P2 receptors [31]. The fact that extracellular ATP at this concentration has little potentiating effect on GABA_A_R currents induced by either exogenous (Figure 1) or endogenous GABA (Figure 2) strongly suggests that activation of purinergic receptors is unlikely to be responsible for the ATP potentiation of GABA currents observed here. To further rule out the potential involvement of activation of P2 receptors, P2 receptor antagonists were perfused prior to, and during the application of ATP. At a concentration of 100 μM, suramin (a broad-spectrum antagonist of P2 receptors) blocks almost all P2 receptors, but has almost no effect on P2X_7_ receptors [32,33]. BBG is a potent P2X_7_ receptor antagonist with a very low IC_50_ (< 10 nM) [34,35]. As shown in Figure 3C, although application of suramin (100 μM) and BBG (1 μM) resulted in reduction of GABA currents on their own, ATP (2 mM) was still able to potentiate GABA currents in the presence of suramin and BBG (Figure 3C). The mean amplitude of GABA currents was increased to 138.5 ± 16.5% (n = 5; P < 0.05) of the control. This suggests that the ATP potentiation of GABA currents is not due to activation of P2 receptors. Together, our results appear to reveal a previously undescribed mechanism of ATP modulation of GABA_A_Rs, one which is not dependent on either ecto-protein kinases or P2 receptor activation.

### The enhancement of GABA_A_R function by extracellular ATP is likely mediated by an allosteric mechanism

**Figure 4:**
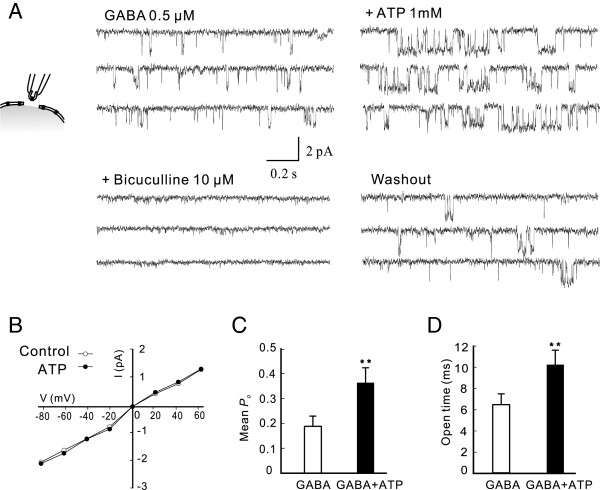
**Extracellular ATP increases the open time of GABA single-channel activity in outside-out mode in hippocampal neurons.** Outside-out GABA_A_R gated single channel activities were recorded by bath application of GABA (0.5 μM) to the excised patches through bath applications (**A**, *Left*) in absence (GABA; 0.5 μM) or presence of ATP (+ATP 1 mM) or ATP + bicuculline (+bicuculline 10 μM) in the bath or after drug washout (Washout). Suramin (100 μM) and BBG (1 μM) were added in the bath throughout the recording to block P2 receptors. **A**, Representative GABA current traces were obtained 5 min before (GABA 0.5 μM) and after drug applications or washout as indicated above traces. In this patch, *P*_*o*_ increased from 0.15 in the control (GABA) to 0.28 after addition of ATP. **B**, Current-voltage relationship of GABA single channel activity in the absence (Control) and presence of ATP (1 mM) at various holding membrane potentials. **C** and **D,** Bar graphs summarizing the mean *P*_*o*_ (*L*eft) and open times (*Right*) in the absence (GABA) and presence of ATP (GABA + ATP) from seven cells. ** P < 0.01.

ATP may also allosterically modulate functions of many proteins, including neurotransmitter receptors, by directly binding to these proteins [17,36-38]. As an initial step to test this possibility, we investigated the effect of extracellular ATP on GABA_A_R-gated single channel activities in excised membrane patches under various modes of single-channel recordings. Suramin (100 μM) and BBG (1 μM) were added in the extracellular solution to block P2 receptors. Under the outside-out mode at a holding potential of –60 mV (Figure 4), no single channel activities were observed in the absence of GABA. ATP alone (1 mM) did not induce any detectable single channel currents (data not shown). When 0.5 μM GABA was added to the extracellular recording solution, single channel activities occurred frequently, but were abolished by the addition of bicuculline (10 μM) in the extracellular solution, confirming that the currents were gated through GABA_A_Rs (Figure 4A). The mean single channel conductance and open probability (*P*_*o*_) were 27 ± 0.85 pS and 0.18 ± 0.04 (n = 7), respectively. As shown in Figure 4A, co-application of ATP (1 mM) significantly increased the *P*_*o*_ of the GABA_A_R-gated single-channel activities without altering either main channel conductance or reversal potential (Figure 4A and B). The mean *P*_*o*_ and single channel conductance in the presence of ATP were respectively 0.32 ± 0.06 (p < 0.01; n = 7; Figure 4C) and 27 ± 1.02 pS (P > 0.05; n = 7). The changes in mean *P*_*o*_ may result from altered channel open times and/or frequency. Open time distribution histograms revealed that the mean open time in the presence of ATP was 11.3 ± 1.5 ms, which was significantly different from the control (6.4 ± 1.1 ms; P < 0.05, n = 7; Figure 4D). Although the frequency in the presence of ATP was slightly increased (control: 2.6 Hz vs. ATP: 3.0 Hz), it was not significantly different from the control (P > 0.05; n = 7). These results suggest that ATP potentiation of GABA currents is achieved by an increase in single channel *P*_*o*_, mainly due to prolonged open times.

**Figure 5:**
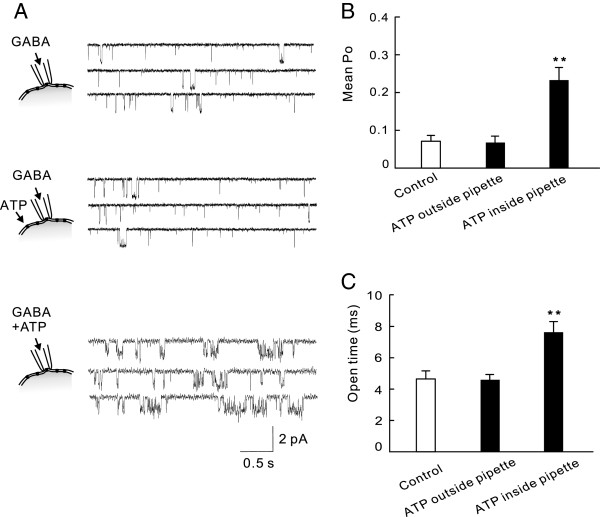
**Extracellular ATP increases GABA single-channel open time in cell attached mode.** GABA_A_R channels in the patch membrane underneath the tip of the recording pipette were specifically activated by inclusion of GABA (0.5 μM) in the intra-pipette recording solution at a holding membrane potential of 0 mV. **A**, Representative current traces revealing that ATP (1 mM) increased the GABA single-channel activity only when it was included through the recording pipette. Bar graphs on the *Right* summarize the mean open probability **(***P*_*o*_; **B)** and open times **(C)** averaged from 5 individual recordings in the absence (Control) and presence of ATP (1 mM) outside the pipette in the bath solution (ATP outside pipette) or inside the pipette by its inclusion in the pipette solution (ATP inside pipette). ** P < 0.01.

The observation that potentiation can be detected in the excised patches under outside-out mode is consistent with the idea that ATP is not dependent on any diffusible intracellular signaling molecule. Thus, ATP may exert its modulating effects by directly acting on the GABA_A_R itself, or on a protein that is tightly associated with the GABA_A_R. This idea was further supported by single-channel recordings under the on-cell attached configuration. In this mode, GABA (0.5 μM) was added to the recording solution in the pipette to selectively activate GABA single channels within the pipette tip (Figure 5A). Bath perfusion of ATP (1 mM) outside of the recording pipette had little effect on single channel activities (Figure 5A and B; the mean *P*_*o*_ and the main channel current amplitude being 0.08 ± 0.02 and 1.66 ± 0.2 pA in the presence of ATP and 0.07 ± 0.02 and 1.64 ± 0.2 pA in the absence of ATP; P > 0.05, n = 5). However, application of ATP (1 mM) along with GABA (0.5 μM) into the recording pipette solution resulted in a significant increase in the mean *P*_*o*_ (*P*_*o*_: 0.19 ± 0.04; P < 0.01, compared with that in the absence of ATP; n = 6; Figure 5A and B) and was similar to that observed under the outside-out mode, leading to a significant increase in the mean open times (Figure 5C; 7.7 ± 0.8 ms in the presence of ATP vs 4.6 ± 0.4 ms in the absence of ATP; P > 0.01, n = 5). Together, single channel results from both outside-out and on-cell modes support the notion that ATP potentiates GABA currents, likely via a mechanism of allosteric modulation.

**Figure 6:**
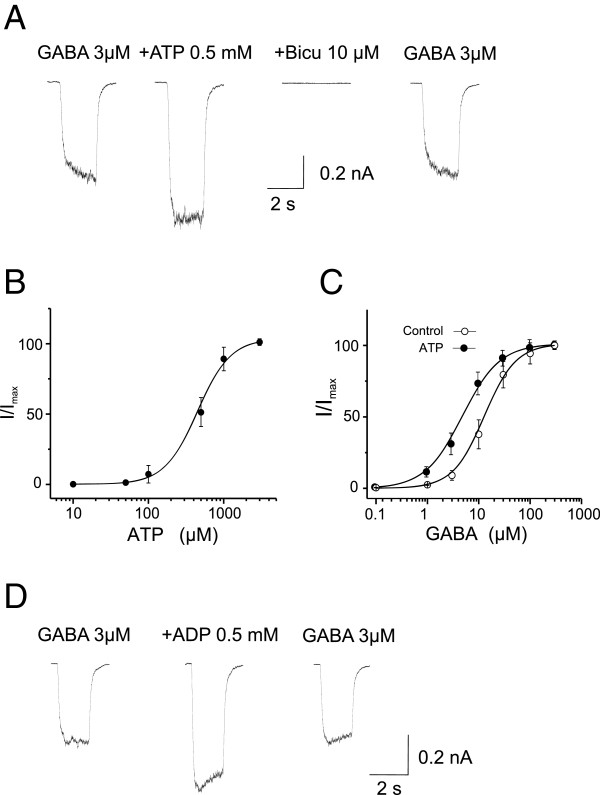
**Extracellular ATP and ADP potentiates function of recombinant GABA**_**A**_**Rs.** Whole-cell voltage-clamp recordings were performed in HEK293 cells transiently expressing rat recombinant α1β2γ2 GABA_A_Rs at a holding membrane potential of -60 mV. GABA currents were induced by fast perfusion of GABA at various concentrations. **A**, Representative current traces showing the potentiation of GABA (3 μM) currents by co-perfusion of ATP (0.5 mM). The increased current was blocked in the presence of bicuculline (10 μM) and returned to the control level after ATP washout. **B**, The dose-response curve showing the dose-dependent potentiation of the GABA (3 μM) currents by various concentration of ATP. All values were normalized to the maximal GABA currents (I_max_) induced at 3 mM of ATP. Each data point is the mean ± SEM of normalized GABA currents at the indicated ATP concentrations (n = 6). The solid line is the best fit of the data to the Hill equation. The EC50 and H were 390 ± 48 μM and 1.66 ± 0.1, respectively. C, ATP potentiation of GABA_A_R activity is GABA dose-dependent. GABA dose-response curves were constructed in the absence (Control) and presence (ATP) of ATP (0.5 mM) from five cells. ATP shifted the curve to the left and reduced EC50 from 11.6 ± 3.3 μM to 5.8 ± 2.1 (P < 0.01) without change in H (control: 1.57 ± 0.1; ATP: 1.62 ± 0.1; P > 0.05). All values were normalized to the I_max_ induced by GABA (300 μM). **D**, ADP mimics ATP, potentiating the activity of recombinant GABA_A_Rs. Representative current traces showed that application of ADP (0.5 mM) reversibly increased currents induced by GABA (3 μM). On average, it increased the currents by 147.5 ± 15.9% (P < 0.01; n = 5).

To further explore this possible allosteric modulation via a direct binding to the GABA_A_R itself, we finally examined the ability of extracellular ATP and its analogs to potentiate the function of recombinant GABA_A_Rs transiently expressed in HEK293 cells in the absence of other known neuronal proteins. GABA_A_Rs are pentameric hetero-oligmers assembled from various distinct subunit combinations. Although more than sixteen distinct subunits have been identified, most native GABA_A_Rs in the mammalian brain consist of 2α, 2β and 1γ subunits [1,39]. We transiently expressed recombinant rat α1β2γ2 GABA_A_Rs, the most abundant composition of native GABA_A_R subtypes in the mammalian brain [39]. Neither ATP nor ADP at a concentration of 2 mM produced any detectable currents in HEK293 cells. Fast perfusion of GABA (3 μM) reliably produced inward currents, which were blocked by 10 μM bicuculline, indicating the currents were mediated by bicuculline sensitive GABA_A_R-mediated chloride-gated ion channels (Figure 6A). Co-perfusion of GABA (3 μM) and ATP at various concentrations resulted in a significant increase in the peak amplitudes in a dose-dependent manner, with a minimum concentration of approximately 100 μM and a maximum concentration of greater than 2 mM (Figure 6A and B; n = 6). At a concentration of 0.5 mM, ATP increased the GABA currents to 138.7 ± 14.2 of the control value (Figure 6A and B; P < 0.05, n = 6). The potentiated currents were completely abolished by bicuculline (10 μM; Figure 6A), and recovered to control levels after washing out ATP (Figure 6A).

This ATP potentiation is also GABA concentration dependent. As shown in Figure 6C, in the absence of ATP, GABA produced currents in a dose-dependent manner with the EC50 and Hill coefficient (*h*) being respectively 11.6 ± 3.3 μM and 1.66 ± 0.1 (n = 5). Bath application of ATP (0.5 mM) caused a left shift of the GABA dose-response curve, reducing the EC50 to 5.8 ± 2.1 μM without altering the *h* (1.62 ± 0.1). Similar to observations obtained in neurons, the ATP potentiation of GABA_A_Rs was also mimicked by ADP. We found that bath application of ADP (2 mM) increased GABA currents by 147.5 ± 15.9% of the control (P < 0.01, n = 5; Figure 5D). Thus, similar to native GABA_A_Rs in neurons, recombinant rat GABA_A_Rs overexpressed in HEK293 cells are also subject to potentiation by extracellular ATP. These results therefore provide additional support for the notion that ATP modulation of GABA_A_Rs through an allosteric mechanism that does not require other neuronal proteins.

## Discussion

The modulation of GABA_A_R function by extracellular ATP observed here is unlikely to require activation of P2Y receptors, as was previously reported in rat cerebellar granule cells [13]. First, Saitow and colleagues found that ADP potentiation of postsynaptic GABA_A_R-mediated currents was long-lasting and could be demonstrated at a much lower ADP concentration (within tens of micromoles). In our study, the potentiating effect of ATP or ADP on GABA currents was transient and reversible, quickly returning to baseline level upon ATP washout. Moreover, ATP potentiation was only observed at concentrations above 100 μM. Second, it is well established that suramin at a concentration of 100 μM can block the majority of P2 receptors (with the exception of P2X_7_ receptors) and that BBG has a very high affinity for the P2X_7_ receptor with an IC50 of 10 nM to 0.2 μM [35]. We found that the potentiating effect of ATP or ADP on GABA currents was not blocked by suramin (100 μM) and BBG (1 μM).

It has been speculated that cross-talk between GABA_A_ and P2X receptors may play in a role in ATP regulation of GABA_A_Rs. Although underlying mechanisms remain controversial, recent studies suggest that it depends on elevated [Ca^2+^]_i_[12,16] (but, also see [16]). However, whether [Ca^2+^]_I_ dependent or not, such a receptor-cross talk is unlikely to be responsible for the ATP potentiation of GABA_A_R function observed in the present study, as blockade of P2 receptors had little effect, and the BAPTA (10 μM) included in our intracellular recording solution should be sufficient to prevent the [Ca^2+^]_I_-dependent processes proposed in these earlier studies.

Ecto-protein kinases have been identified in the CNS and can modulate functions of membrane receptors such as P2X3 receptors [30]. However, in the present study we demonstrated that both AMP-PNP and ADP mimicked the effects of ATP, potentiating the function of GABA_A_Rs. As both of AMP-PNP and ADP cannot substitute ATP in supporting the protein phosphorylation reaction, these results can essentially rule out the involvement of ecto-protein kinase mediated ex extracellular protein phosphorylation.

ATP is also known to function as an allosteric modulator for a number of proteins by directly binding to these proteins, regulating their functions. These proteins include CFTR (Quinton PM, Reddy MM., 1992), GABA_A_Rs [40], InsP3 receptors [37,41], and capsaicin-activated ion channels [42]. But, in most of these cases, ATP binds to the intracellular domains of these proteins. Whether extracellular ATP can allosterically modulate GABA_A_Rs via a direct binding to the extracellular domains of the receptor has not previously been suggested. Here, we provide several pieces of evidence that are consistent with such a mode of regulation. First, using recombinant rat GABA_A_Rs transiently expressed in HEK293 cells, we were able to demonstrate that extracellular ATP can potentiate the function of these recombinant GABA_A_Rs in a manner similar to observations obtained with native GABA_A_Rs in neurons, suggesting that the ATP modulation does not require any additional neuronal specific proteins other than GABA_A_Rs themselves. Second, ATP potentiation could be demonstrated in the excised cell-free membrane patches under the outside-out configuration in neurons (Figure 4). These results indicate that the potentiation of GABA_A_Rs by extracellular ATP does not require any diffusible second messenger molecule downstream of an unknown metabotropic receptor, thereby providing strong support for a direct binding of ATP or its analog to an extracellular domain of GABA_A_R itself, or a membrane surface protein tightly associated with the receptor. Finally, this notion is further strengthened by our results obtained with single-channel recordings under the on-cell attached configuration (Figure 5). Under this configuration, currents through single or very few GABA_A_R channels in the membrane patch underneath of the tip of the recording pipette can be recorded in isolation from GABA_A_Rs outside of the pipette tip by applying GABA to the isolated membrane patch through the recording pipette solution. Using this configuration, we were able to demonstrate that the GABA_A_R single channel activities from the isolated membrane patch inside of the recording pipette can only be potentiated by ATP applied into the patch membrane through the recording pipette solution, but not by that applied extracellularly to the plasma membrane outside of the recording pipette tip (Figure 5). Together, the present study provides strong evidence supporting a novel mode of modulation of GABA_A_Rs by extracellular ATP; allosteric modulation likely achieved by either a direct binding of ATP to the receptor itself, or to an unknown protein tightly associated with the receptor. Nonetheless, given that extracellular ATP may potentially penetrate the plasma membrane, the possibility that ATP potentiates GABA_A_R function by a direct action on an intracellular domain of the receptor remains to be ruled out. Thus, the ultimate evidence for such a novel allosteric modulation will only come from the positive identification/characterization of the novel binding site (s) on GABA_A_R domain (s) or to identify the receptor associated protein by which ATP acts. In addition, whether the potentiation of GABA_A_Rs by ATP is state-dependent remains to be determined.

How ATP binding modulates the GABA_A_R remains to be determined. ATP binding may cause a conformational change to the GABA_A_R, thereby affecting its agonist binding affinity, channel gating, or both. Using various modes of single-channel recordings and analysis, in the present study we observed that extracellular ATP can increase GABA_A_R-gated channel activities by primarily increasing the channel open times without altering its conductance. This may suggest that ATP binding predominantly alters the apparent agonist binding affinity to the receptor, rather than the conformational change of the channel pore. This conjecture is further strengthened by the GABA dose-response relationship analysis from HEK293 cells as extracellular ATP results in a left-shift of the GABA dose-response curves without altering either *h* coefficient or the maximal responses (Figure 6B). Future site-direct mutations of the putative ATP binding domain along with direct ATP binding assays may provide a better understanding of the detailed mechanisms by which ATP exerts its modulation of the GABA_A_R.

In the mammalian brain, GABA_A_Rs play a key role in regulating the excitation-inhibition balance (and hence the tight control of neuronal excitability), and this function is primarily realized by mediating synaptic (phasic) inhibition and tonic inhibition [26]. In the present study, we demonstrated that application of extracellular ATP at millimolar concentrations not only potentiates GABA currents evoked by exogenously applied GABA, but also both synaptic and tonic currents activated by endogenous GABA. These results suggest that extracellular ATP has significant physiological and/or pathologic impacts on neuronal excitability via modulating GABA_A_Rs. To this end, it is relevant to point out that several previous studies have suggested that under normal conditions, the extracellular ATP in the CNS is approximately 1-100 μM [25]. At such a low level, the basal extracellular ATP may have little influence on neuronal excitability via GABA_A_Rs. However, it is important to note that ATP has previously been shown to be co-localized with GABA in the same vesicles at certain GABAergic synapses, and more importantly, that these two transmitters can be co-released into the synaptic cleft [18,19,25], whereby ATP concentrations can transiently reach the levels above hundreds of micromoles or even millimolar concentrations. In the present work, we demonstrated that at these concentrations, extracellular ATP can potentiate GABA_A_R-mediated mIPSCs. The fact that ATP potentiation of mIPSCs is primarily manifested as a specific increase in mIPSC amplitude, without altering its frequency, is in good agreement with the allosteric modulation of postsynaptic GABA_A_Rs. Thus, this mode of modulation may function at certain GABAergic synapses under physiological conditions. Similarly, the modulation may also occur under pathological conditions (including neuronal overexcitation, epileptic episodes, inflammation, traumatic insults, hypoxia/ischemia), as ATP release from damaged neurons and astrocytes can rapidly increase extracellular ATP concentrations [21-24]. ATP may increase both phasic and tonic GABA currents by acting on both synaptic and extrasynaptic GABA_A_Rs. By forming such a homeostatic feedback loop, under pathological conditions extracellular ATP may exert significant impacts on neuronal function and/or dysfunction. However, a related caveat is the potential complication from acidosis that is often associated with high concentrations of extracellular ATP. Given that acidosis is known to reduce GABA_A_R activity, how it will impact this ATP-induced allosteric potentiation warrants future investigations.

## Conclusions

In this study, we demonstrate that extracellular ATP and its analogs such as ADP can potentiate function of GABA_A_Rs via a novel mechanism likely involving the direct binding of ATP to a putative ATP-binding site on the GABA_A_R itself. We demonstrate that through this modulation, extracellular ATP can enhance both phasic (synaptic) and tonic GABA_A_R-mediated currents. Therefore, the present study reveals a novel means by which extracellular ATP contributes to regulating excitation-inhibition balance and neuronal excitability under certain physiological and pathological conditions. In addition, due to the importance of GABA_A_Rs in mediating neuronal inhibition in the brain, they have been a major therapeutic target for the development of many drugs currently used for the clinical treatment of brain disorders. Further identifying the exact putative binding site of ATP on GABA_A_Rs may lead to the development of novel GABAAR-based therapeutics for better management of these brain disorders.

## Materials

### Primary culture of hippocampal neurons

Methods for culturing hippocampal neurons have been described previously [43]. Briefly, hippocampi from E18 old Wistar rat embryos were dissected and treated with 0.25% trypsin solution (Invitrogen) for 25 min at 37°C, then mechanically dissociated using fire-polished pasteur pipettes. Cell suspension was centrifuged at 2500 × *g* for 50 s and the cell pellets were resuspended in DMEM with 10% fetal bovine serum (FBS). Cells were seeded on poly-D-lysine-coated 24-well coverslips at a density of 0.8-1.0 × 10^5^ cells/well. Cultures were maintained in a humidified incubator with 5% CO_2_ at 37°C. After 24 h, the plating medium was changed to Neurobasal medium supplemented with B-27 and L-glutamine (0.5 mM) and neurons were fed with fresh medium twice weekly. Experiments were done 14-18 days after the plating.

### Expression of recombinant GABA_A_ receptors in HEK293 cells

Human embryonic kidney (HEK) 293 cells were cultured as previously described [44]. Briefly, HEK293 cells were cultured in DMEM supplemented with 10% FBS. Cells were harvested weekly and seeded at 10% confluence on poly-L-lysine-coated glass coverslips in 24-mm culture dishes. Cells were transiently co-transfected at 70% confluence with rat cDNAs encoding α1, β2 and γ2-EGFP subunits of the GABAA receptor at a 1:1:1 ratio using Lipofectamine2000 (Invitrogen, Carlsbad, CA). Recordings were made 24-48 h after transfection.

### Whole-cell patch clamp recording

Coverslips were transferred to the recording chamber and were continuously perfused with an extracellular solution containing the following (in mM): NaCl 140, KCl 5.4, MgCl_2_ 1.3, HEPES 25, CaCl_2_ 1.3, glucose 20, pH 7.35-7.45, 305-315 mOsm. Recordings were performed in the voltage-clamp mode using an Axopatch 200B patch-clamp amplifier (Axon Instruments). The cell membrane were held at a potential of -60 mV and signals were filtered at 2 KHz, digitized at 10 KHz using a Digidata 1322A analog-to-digital converter and acquired by Clampex 9.2 (Axon Instruments). Recording electrodes (3–5 M) were fabricated from thin-walled borosilicate glass tubing (World Precision Instruments, USA) with a micropipette puller (Sutter Instruments, model P-97, Novato, CA). Recording pipettes were filled with an intracellular solution containing (in mM): CsCl 140, HEPES 10, 1,2-*bis* (2-aminophenoxy) ethane-*N, N, N, N*-tetraacetic acid (BAPTA-Cs) 10, Mg-ATP 4, QX-314 5, pH 7.20; osmolarity, 290-295 mOsm. CNQX (20 μM), AP-5 (50 μM) and tetrodotoxin (TTX, 0.5 μM) were included in the external solution to block glutamatergic and the voltage-gated sodium channels. All experiments were performed at room temperature.

### Induction of GABA_A_R-mediated currents

Glass pipettes were filled with GABA (100 μM) dissolved in the extracellular recording solution. The pipette tip was placed in the vicinity of recorded neurons. GABA was applied via pressure ejection using a Picospritzer (General Valve Corporation, Fairfield, NJ) at 60 sec intervals. For recording of GABA currents in HEK293 cells, fast perfusion of GABA and/or other ligands were employed using a computer-controlled multibarrel fast perfusion system (Warner Instruments). For some of the experiments using bath perfusion of ATP or ADP, 5 mM EGTA was added into Ca^2+^-free extracellular solution to further reduce residual Ca^2+^ in extracellular solution, thereby minimizing any potential effect of ATP/ADP-induced extracellular Ca^2+^ influx. Under our experimental conditions in cultured neurons, the Ca^2+^-free solution neither produced any observable current on its own nor significantly affected GABA_A_R-gated currents (1 μM GABA; -648 ± -245pA in control solution vs -677 ± -256pA in Ca^2+^-free solution supplemented with 5 mM EGTA; n = 7; p < 0.05).

### mIPSCs and GABA tonic current recording and analysis

GABA_A_ receptor-mediated miniature inhibitory postsynaptic currents (mIPSCs) were recorded at a holding potential of −60 mV. CNQX (20 μM), APV (50 μM) and TTX (1 μM) were added to the extracellular solution to isolate GABAergic mIPSCs. Before drug application, a 3-5 min period of baseline recording (control) was obtained. The recordings were low-pass filtered (Clampfit software) at 2 kHz, digitized at 10 KHz using a Digidata 1322A analog-to-digital converter and acquired by Clampex 9.2. Detection and analysis of mIPSCs were performed using Mini Analysis Program (Synaptosoft, Decatur, GA). Any spurious noise was rejected. The mIPSC kinetics were obtained from analysis of the averaged single events. To facilitate analysis, decay time constant (τ_D_) was obtained by fitting the decay phase to a single exponential equation. Tonic GABA current was estimated as the change in baseline current produced by a 2 min application of bicuculline (20 μM).

### Single channel analysis

Patch pipettes for cell-attached and outside-out single channel recordings were pulled from thick-wall borosilicate glass (GC150F, Harvard Apparatus), fire polished, and coated with Sylgard 184 (Dow Corning) with a resistance of 6-10 MΩ. For outside-out recordings, the recording pipette was filled with the intracellular solution at a holding potential of – 60 mV. GABA (0.5 μM) was added to the external solution to activate GABA_A_Rs. In cell-attached patch mode, the composition of the pipette solution was (in mM): KCl 120, TEA-Cl 20, 1.3 MgCl_2_, HEPES 10, pH 7.40, 290-300 mOms. GABA (0.5 μM) or GABA (0.5 μM) and ATP (1 mM) were included in the pipette solution. Signals were filtered at 1 kHz, sampled at 10 kHz and analyzed off-line using Clampfit 9.2. An idealized recording of the durations and amplitudes of detectable events of the single-channel data was generated using 50% threshold crossing criteria. Events with a duration less than 300 μs were ignored. Single channel activities were expressed as the product of the number of channels × the open probability (*P*_*o*_); i.e. NP_o_ = ∑[(open time × number of channels open) ∕ total time of record].

### Drugs

Drugs used in the present study were purchased from the following sources: nucleotides (ATP, UTP, GTP, ADP, UDP, GDP, AMP, UMP, GMP), GABA and bicuculline (Sigma-Aldrich); suramin, CNQX, APV, PPADS (Tocris); and TTX (Alomone Labs).

### Statistical analysis

Data is presented as means ± SEM, where n represents the number of tested cells. One-way ANOVA or the two-tailed Student’s test was used for statistical analysis and P values less than 0.05 were considered statistically significant. Dose–response curves were constructed by fitting data to the Hill equation: *I* = *I*_*max*_/(1 + EC_50_ / [A]^n^), where *I* is the current, *I*_*max*_ is the maximum current, [A] is a given concentration of agonist, n is Hill coefficient (H).

## Competing interests

The authors declare that they have no competing interests.

## Authors’ contributions

JL and YTW designed the experiments. JL performed and analyzed all experiments. JL and YTW wrote the manuscript. Both authors read and approved the final manuscript.
